# Predicting Ca^2+^ and Mg^2+^ ligand binding sites by deep neural network algorithm

**DOI:** 10.1186/s12859-021-04250-0

**Published:** 2022-01-20

**Authors:** Kai Sun, Xiuzhen Hu, Zhenxing Feng, Hongbin Wang, Haotian Lv, Ziyang Wang, Gaimei Zhang, Shuang Xu, Xiaoxiao You

**Affiliations:** 1 College of Sciences, Inner Mongolia University of Technology, Hohhot, 010051 People’s Republic of China; 2 Inner Mongolia Key Laboratory of Statistical Analysis Theory for Life Data and Neural Network Modeling, Hohhot, People’s Republic of China; 3 College of Data Science and Application, Inner Mongolia University of Technology, Hohhot, 010051 People’s Republic of China; 4 Hohhot First Hospital, Hohhot, 010051 People’s Republic of China

**Keywords:** Deep learning algorithm, Protein, Metal ion ligand, Binding residue

## Abstract

**Background:**

Alkaline earth metal ions are important protein binding ligands in human body, and it is of great significance to predict their binding residues.

**Results:**

In this paper, Mg^2+^ and Ca^2+^ ligands are taken as the research objects. Based on the characteristic parameters of protein sequences, amino acids, physicochemical characteristics of amino acids and predicted structural information, deep neural network algorithm is used to predict the binding sites of proteins. By optimizing the hyper-parameters of the deep learning algorithm, the prediction results by the fivefold cross-validation are better than those of the Ionseq method. In addition, to further verify the performance of the proposed model, the undersampling data processing method is adopted, and the prediction results on independent test are better than those obtained by the support vector machine algorithm.

**Conclusions:**

An efficient method for predicting Mg^2+^ and Ca^2+^ ligand binding sites was presented.

## Background

The combination of protein and alkaline earth metal ion ligands affects many physiological processes in the human body. For example, vascular smooth muscle must combine with Mg^2+^ to play the role of dilating blood vessels and regulating blood pressure [1], and thrombin in blood must combine with Ca^2+^ to perform in coagulation and hemostasis [2]. A large number of studies predicted the binding residues of protein-alkaline earth metal ion ligands. But metal ion ligands are small, active and hard to be predicted, which leads to a generally large false positive in the research results. Therefore, the study of protein-alkaline earth metal ion ligand binding residues is challenging.

Uneven positive and negative data size limits the improvement of prediction accuracy. Generally, there are two kinds of data processing methods: one is to eliminate data imbalance between positive and negative by putting different weight on them. For example, in 2005, Lin et al. [3] used artificial neural network (ANN) method to predict Ca^2+^ ligand binding residues. In 2016, Hu et al. [4] developed a method called Ionseq to predict Ca^2+^ and Mg^2+^ ion ligands. In 2016, Jiang et al. [5] used support vector machines (SVM) algorithm to predict Ca^2+^ ligand binding residues. The other method is to process the data set with undersampling. We selected negative segments with the equal number of positive segments from non-binding fragments to compose negative set to construct the data set with equal number of positive and negative sets for prediction. For example, in 2017, Cao et al. [6] used SVM algorithm to predict the binding residues of Ca^2+^ and Mg^2+^ ion ligands. In 2019, Wang et al. [7] predicted the ligand binding residues of Ca^2+^ and Mg^2+^ ions by using sequential minimal optimization algorithm. In 2020, Hu et al. [8] used Gradient Boosting Machine algorithm to predict Ca^2+^ and Mg^2+^ ion ligand binding residues.

In terms of algorithms, many machine learning algorithms have been widely used in the prediction of protein-metal ion ligand binding residues. For example, in 2004, Sodhi et al. [9] identified Ca^2+^ and Mg^2+^ ion ligand binding residues based on ANN. In 2006, Lin et al. [10] predicted the binding residues of Ca^2+^ and Mg^2+^ ion ligands by using SVM algorithm. In 2010, Horst et al. [11] predicted Ca^2+^-binding residues in proteins by multiple sequence comparison analysis. In 2012, Lu et al. [12] used the "fragment transformation" method to predict Ca^2+^ and Mg^2+^ ion ligand binding residues. In 2020, Liu et al. [13] used random forest (RF) algorithm to predict the binding residues of Ca^2+^ and Mg^2+^ ion ligands. Among various algorithms, the RF algorithm and SVM algorithm have relatively good prediction results. Although the Sp, ACC and MCC values obtained by the RF algorithm are high, the Sn values are low. While, SVM has more balanced performance. Sp, ACC and MCC values of SVM are slightly lower than those of RF algorithm, but SVM is outstanding on sensitivity and reduces the number of false positives. It is more likely to predict the binding residues correctly in actual prediction. Overall, among traditional machine learning algorithms, SVM algorithm has better prediction performance.

Since the rise of deep learning algorithms in 2012, breakthroughs are made in natural language, speech processing, machine translation and other fields [14–16] of big data. There is a certain similarity between the processing of the natural language problem and the prediction of protein binding residue. Studies showed [17] that performing the frequency analysis on amino acids, their distribution obey Zipf's law, which was considered to be one of the fundamental features of language. This meant that biological sequences can be considered as "natural language" existing in nature and suitable for deep learning algorithms. For example, in 2019, Cui et al. [18] used the Deep convolutional networks algorithm, based on entire amino acid sequences, controlled the size of the effective context scope by the number of convolution layers, and captured the local information of binding residues and the long-distance dependence between amino acids layer by layer to predict the binding residues of six metal ion ligands. While we have processed the protein sequence into shorter amino acid fragments, and controlled the size of the effective context scope. Therefore, we adopted a more concise deep learning algorithm, i.e., deep neural networks (DNN) algorithm. It is built through the fully-connected layers, expresses the essential information contained in the data through multi-layer nonlinear variation, and reduces the dimension of high-dimensional data, so that it can learn more effective features.

Therefore, in this paper, DNN algorithm was used to predict Ca^2+^ and Mg^2+^ ligand binding residues, and the results of fivefold cross-validation were better than those of Ionseq method [4] after optimization of hyper-parameters. To further verify the performance of the proposed model, we used the method of undersampling to deal with the data set. By optimizing parameters, we adopted fivefold cross-validation and independent tests. The independent test results were better than those of SVM algorithm. The research showed that: DNN algorithm has certain advantages in predicting Ca^2+^ and Mg^2+^ ligand binding residues.

## Methods

### Establishment of dataset

To ensure the authenticity of the data and the accuracy of the experiment, we selected the data from BioLip database [19] and downloaded protein chain that interacts with Ca^2+^ and Mg^2+^ ligands. BioLip database is a semi-manual database, and the data are measured accurately by experiments. To build a non-redundant dataset, we filtered the data and eliminated protein chains with the sequence length of less than 50 amino acids, the resolution of more than 3 Å, and the sequence identity greater than 30%. Compared with Hu et al. [4], the amount of non-redundant data set obtained is obviously increased. The number of protein chains interacting with Ca^2+^ ligand increases from 179 to 1237, and Mg^2+^ ligand increases from 103 to 1461.

When a protein combines with a metal ion ligand, both the binding residues and the surrounding residues will be affected. In order to extract more comprehensive information, we used the sliding window method to intercept fragments on protein sequences, and the length *L* of the intercepted fragments was taken as 9 according to references [6, 7] for the ligands of Ca^2+^ and Mg^2+^. To ensure that every amino acid appears in the center of the fragment, we added (*L* − 1)/2 pseudo amino acids at both ends of the protein chain. If the fragment center was a binding residue, it would be defined as a positive set fragment, otherwise it would be a negative set fragment. The data set of alkaline earth metal ion ligands obtained is shown in Table 1. It can be seen from the data in Table 1 that the fragments of negative set are much larger than those of positive set, the number of fragments of Ca^2+^ ligand negative set is more than 58 times that of positive set, and that of Mg^2+^ ligand negative set is more than 92 times that of positive set.

**Table 1:** Alkaline earth metal ion ligand data set

Ligands	Data set	Chains	P	N
Ca^2+^	OUR’S	1237	6789	396,957
	HU’S [4]	179	1360	119,192
Mg^2+^	OUR’S	1461	5212	480,307
	HU’S [4]	103	391	76,382

Note: Ligand represents the metal ion ligand; Chains represents the number of protein chains combined with metal ion ligands; P represents the binding residue of metal ion ligand; N represents the non-binding residue of metal ion ligand

### Selection of characteristic parameters

Based on the sequence of amino acids, this paper selected amino acids, physicochemical characteristics of amino acids and predicted structural information as characteristic parameters. Among them, the physicochemical characteristics of amino acids included the charge and hydrophobicity of amino acids. According to the charge properties of amino acids, 20 kinds of amino acids can be divided into 3 categories [20]. Amino acids K, R and H were positively charged, D and E were negatively charged, and other amino acids were not charged. According to the hydrophilic and hydrophobic properties of amino acids, 20 kinds of amino acids were divided into 6 categories [21]. The amino acids R, D, E, N, Q, K and H were strongly hydrophilic, L, I, V, A, M and F are strongly hydrophobic, S, T, Y and W were weakly hydrophilic, and P G and C each belongs to one category. The predicted structural information included secondary structural information, relative solvent accessibility area and dihedral angle (phi angle and psi angle), all of which were obtained from the prediction of protein sequences by the ANGLOR [22] software. The secondary structure information included three types: α-helix, β-fold and random curl. Based on statistical analysis, the area information of solvent accessibility was divided into four intervals [6], *x* represented the value of relative solvent accessibility area and its threshold was expressed by *r*(*x*):

1 $$r(x) = \left\{ \begin{gathered} I,x \in (0,0.2] \hfill \\ II,x \in (0.2,0.45] \hfill \\ III,x \in (0.45,0.6] \hfill \\ IV,x \in (0.6,0.85] \hfill \\ \end{gathered} \right.$$

The dihedral angle information was reclassified in line with statistics [13], *x* represented the angle of the dihedral angle, the threshold value of phi angle was expressed by function *g*(*x*), and the threshold value of psi angle was expressed by function *h*(*x*):

2 $$g(x) = \left\{ {\begin{array}{*{20}l} {I{\text{ }},{\text{ x}} \in [ - 180^{ \circ } , - 75^{ \circ } ]} \hfill \\ {II,{\text{ x}} \in ( - 75^{ \circ } ,180^{ \circ } ]} \hfill \\ \end{array} } \right.$$

3 $$h(x) = \left\{ {\begin{array}{*{20}l} {I{\text{ }},{\text{x}} \in [ - 180^{ \circ } ,15^{ \circ } ]} \hfill \\ {II{\text{ }},{\text{x}} \in (15^{ \circ } ,135^{ \circ } ]} \hfill \\ {III,{\text{x}} \in (135^{ \circ } ,180^{ \circ } ]} \hfill \\ \end{array} } \right.$$

### Extraction of feature parameters

#### Extraction of component information

We extracted from each fragment for the following component information (37 dimensions):

1. The frequency of occurrence of amino acids to obtain 21-dimensional amino acid composition information.
2. The frequency of occurrence of three secondary structures corresponding to amino acids to obtain 4-dimensional secondary structure composition information.
3. The frequency of 4 relative solvent accessibility area classifications corresponding to amino acids to obtain 5-dimensional relative solvent accessibility information.
4. The frequency of occurrence of 2 phi angles classifications corresponding to kinds of amino acids to obtain 3-dimensional phi angle component information. Similarly, the psi angle is counted to obtain 4-dimensional psi angle component information.

#### Conservative characteristics of loci

We used the position weight matrix [23, 24] to extract the conservative features of sites, and the matrix element of the position weight matrix were expressed as follows:

4 $$m_{{i,j}} = \ln \left( {\frac{{p_{{i,j}} }}{{p_{{0,j}} }}} \right)$$

The pseudo-counting probability *P*_*i,j*_ is expressed as:

5 $$p_{{i,j}} = \frac{{\left( {n_{{i,j}} + \frac{{\sqrt {N_{i} } }}{q}} \right)}}{{\left( {N_{i} + \sqrt {N_{i} } } \right)}}$$

In the formula, *P*_*0,j*_ represents the background probability, and *P*_*i,j*_ represents the occurrence probability of the *j*th amino acid at the *i*th site. *n*_*i,j*_ represents the frequency of the *j* amino acid at the *i* site, *N*_*i*_ represents the total number of amino acids at the *i* site, and *j* represents 20 kinds of amino acids and vacancies. *q* represents the classification number, here 21. Two standard scoring matrices can be constructed from the positive and negative training sets, and each segment can obtain 2L-dimensional feature vectors. Similarly, the predicted secondary structure, relative solvent accessibility area and dihedral angle (phi angle and psi angle) can also be extracted by this method, where *q* is 4, 5, 3 and 4 respectively.

Finally, we got the information of site conservation in each fragment (2L*5 dimensions):

1. 2L-dimensional position conservation information of 20 amino acids.
2. 2L-dimensional position conservation information of 3 secondary structures.
3. 2L-dimensional position conservation information of 4 relative solvent accessibility.
4. 2*2L-dimensional position conservation information of phi and psi angle.

#### Information entropy

For the physicochemical characteristics of amino acids, we used information entropy [13, 25] to extract them in order to avoid the information "overwhelming" caused by imbalanced classification.

The information entropy formula is expressed as:

6 $$H(x) = - \sum\limits_{{j = 1}}^{q} {p_{j} \log _{2} p_{j} }$$

7 $$p_{j} = \frac{{_{{\mathop n\nolimits_{j} }} }}{N}$$

In which *p*_*j*_ represents the probability of occurrence of the *j*th classification in a segment, *n*_*j*_ represents the frequency of occurrence of the *j*th classification in a segment, and *N* is the segment length. For the value of *q*, if it represents charge classification, *q* = 3; if it represents the classification of hydrophilic and hydrophobic, *q* = 6. Finally, we got the one-dimensional information entropy of hydrophilic and hydrophobic water and the one-dimensional information entropy of charge information.

### Deep learning algorithm

Inspired by biological neural network, deep learning algorithm combines low-level features to form a deep neural network with abstract representation, and then simulates the thinking of human brain for perception, recognition and memory, so as to realize high-level feature extraction and expression of complex structural data containing complex information [26]. DNN is one of the common deep learning methods, and its multi-layer network structure expands the neural network's ability to process complex data, processing big data effectively. The protein chain with Ca^2+^ and Mg^2+^ ligands contains hundreds of thousands of fragments of positive set and negative set, and its data amount is suitable for deep learning algorithm. Therefore, this paper choosed DNN algorithm as the prediction tool.

The deep learning algorithm modules used in this paper are all implemented in the keras framework:

1. The normalization module was used to standardize the data to improve the convergence speed and robustness of the training process.
2. The Earlystop module was used to reduce invalid time cost. If Epoch precision did not rise for 10 consecutive times, it was considered that the best precision has been achieved, and training was stopped to prevent over-fitting.
3. The Relu function is used as the hidden layer activation function, and the Sigmoid function as the output layer activation function.

## Results

### Evaluation index

For the evaluation of prediction results, we used the methods commonly used in prediction research of protein-metal ion ligand binding residue [5, 7, 8]: sensitivity (Sn), specificity (Sp), accuracy (Acc), Matthew’s correlation coefficient (MCC). The expressions are:

8 $$S_{n} = \frac{{TP}}{{TP + FN}} \times 100\%$$

9 $$S_{P} = \frac{{TN}}{{TN + FP}} \times 100{\text{\% }}$$

10 $$A{\text{cc}} = \frac{{TP + TN}}{{TP + TN + FP + FN}} \times 100{\text{\% }}$$

11 $$MCC = \frac{{(TP \times TN) - (FP \times FN)}}{{\sqrt {(TP + FP)(TP + FN)(TN + FP)(TN + FN)} }}$$

In which TP represents the number of metal ion ligand binding residues correctly identified; FN represents the number of metal ion ligand binding residues identified as metal ion ligand non-binding residues; TN represents the number of non-binding residues of metal ion ligands correctly identified; FP is the number of metal ion ligand non-binding residues identified as metal ion ligand binding residues.

### The prediction results of fivefold cross-validation

Based on the characteristics parameters of secondary structure, relative solvent accessibility area, dihedral angle, charge and hydrophilicity as characteristic parameters, DNN algorithm was used to predict the binding sites. In the results of fivefold cross-validation, the Sn value of Ca^2+^ and Mg^2+^ ligands reached 13.1%, Sp and Acc value reached 97.1%, MCC value reached 0.115, and the predicted results were not ideal. Therefore, in order to further improve the prediction accuracy, we optimized the DNN algorithm with hyper-parameters.

#### Optimization of hyper-parameters

The hyper-parameters of deep learning algorithm include: the number of hidden layers, learning rate, the number of hidden layer nodes and batch sizes, etc. The hyper-parameters has great influence on the training and performance of the model. Therefore, we can optimize the hyper-parameters and select a group of hyper-parameters with the best prediction results, so as to improve the performance of the algorithm. When optimizing a certain kind of hyper-parameters, other hyper-parameters remained unchanged, then the exhaustive method was used in the range of hyper-parameters, and finally a group of parameters with the best prediction performance in the test set was selected. Considering the influence on model accuracy, computing resources and computing time, referring to previous studies [27, 28], we selected three hyper-parameters, namely, the number of hidden layers, the number of hidden layer nodes and the batch size, to optimize, and gave the value range of optimized hyper-parameters, as shown in Table 2.

**Table 2:** Value range of the hyper-parameter in DNN

Hyper-parameters	Value
Hidden layers	1, 2, 3, 4, 5, 6, 7, 8
Hidden neurons	2, 4, 8, 16, 32, 64, 128
Batch size	2, 4, 8, 16, 32, 64, 128

##### The impact of changes in the number of hidden layers on the prediction accuracy

**Fig. 1:**
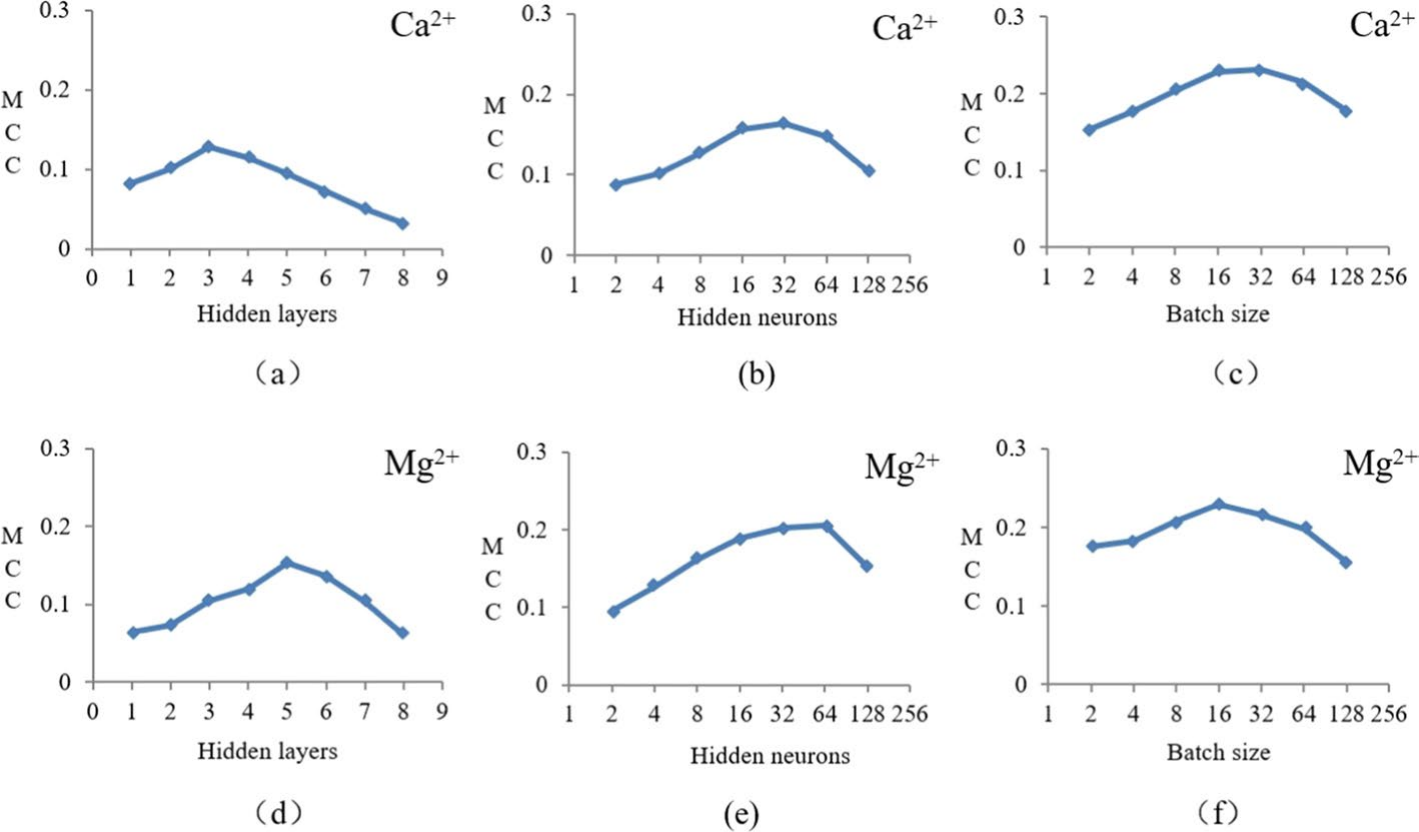
Curve of MCC value of Ca^2+^ and Mg^2+^ ligands with hyper-parameters

Hidden layer is the network layer between the input layer and the output layer, which has the greatest and most intuitive influence on the network structure, and its number of layers can be adjusted by the feedback of prediction results. Setting the number of hidden layer nodes and batch size as fixed values, the number of hidden layers is increased from 1. The results are shown in Fig. 1.

Figure 1a is a line chart showing the MCC value of Ca^2+^ ligand changing with the number of hidden layers. With the increase of the number of layers, the MCC value of Ca^2+^ ligand gradually increased, and reached the highest point of 0.128 when the number of layers was 3, while the MCC value continued to decrease when the number of layers continued to increase. At the same time, referring to the other three evaluation indexes, it can be determined that the optimal layer value of Ca^2+^ ligand is 3. Similarly, Fig. 1d is a line chart showing the MCC value of Mg^2+^ ligand changing with the number of hidden layers. It can be seen from the figure that the optimal layer value of Mg^2+^ ligand is 5.

##### The influence of the change of the number of hidden layer nodes on the prediction accuracy

The number of hidden layer nodes need to be adjusted according to the actual situation of the data set. When the number of hidden layer nodes was small, it will be difficult for the network to learn features effectively. Too much hidden layer nodes will increase the complexity of the network structure and reduce the learning speed of the network, which will also lead to over-fitting. Like the process of optimizing the number of hidden layers, we fixed the number of hidden layers and batch size, and then changed the number of hidden layer nodes to find the optimal value. See Fig. 1b, e for the line chart of MCC value changing with the number of nodes. It can be seen that the optimal hidden layer node value of Ca^2+^ ligand is 32, and Mg^2+^ ligand is 64.

##### The impact of batch size changes on prediction accuracy

The value of batch is the number of samples input for training once. Batch size has obvious influence on the data processing and convergence speed of the algorithm. In the previous article, the optimal number of hidden layers and hidden layer nodes had been determined, so we directly optimized the batch size under these two optimal parameters. See Fig. 1c, f for the line chart of MCC value changing with batch size. It can be seen that the optimal batch size of Ca^2+^ ligand was 32 and Mg^2+^ ligand was 16.

Finally, we got the optimized hyper-parameters and the optimized prediction results, as shown in Table 3.

**Table 3:** Comparison of fivefold cross-validation results

Ligand	hyper-parameter tuning	Hidden layers	Hidden neurons	Batch size	Sn (%)	Sp (%)	Acc (%)	MCC
Ca^2+^	DNN (optimized)	3	32	32	26.4	98.6	97.4	0.231
	IonSeq [4]	–	–	–	22.7	99	98.2	0.211
Mg^2+^	DNN (optimized)	5	64	16	32.8	98.3	97.6	0.229
	IonSeq [4]	–	–	–	5.6	99.9	99.5	0.183

In order to verify the reliability and practicability of DNN algorithm, we also compared it with the results of Ionseq method [4], and the results of Ionseq method were also listed in Table 3.

### Prediction results based on undersampling method

In order to reduce the influence of data imbalance, we also adopted the method of undersampling [22] to process the data set, and randomly selected the negative sequence fragments equal to the positive set; In order to ensure the stability of the prediction results, the negative set samples were randomly selected 10 times, and the average of the 10 results was taken as the final prediction result. Because the data set constructed by the undersampling method can not accurately simulate the actual forecast situation, we also constructed an independent test data set. The metal ion ligand binding protein chain was divided into two parts: one part accounted for 80% of the total protein chain number, which was used as the training set for the network model, and the other part accounted for 20%, which was used as the independent test set. See Table 4 for the independent test data set of alkaline earth metal ion ligands.

**Table 4:** Independent test data set

Ligand	Training dataset			Independent testing dataset		
	Chains	P	N	Chains	P	N
Ca^2+^	989	5256	312,876	248	1533	84,081
Mg^2+^	1168	4069	384,365	293	1143	95,942

Based on the characteristics parameters of secondary structures, relative solvent accessibility area, dihedral angle, charge and hydrophilic-hydrophobic as characteristic parameters, DNN algorithm was used to predict the binding sites. The results of fivefold cross-validation using training dataset are shown in Table 5. The independent testing dataset was input into the prediction model after the optimization of the hyper-parameter, and the prediction results of independent testing were shown in Table 5.

**Table 5:** Comparison of results of DNN algorithm and SVM algorithm

Ligands	Algorithms	Hidden layers	Hidden neurons	Batch size	Sn (%)	Sp (%)	Acc (%)	MCC
Ca^2+^	DNN^①^	2	16	32	80.1	74.6	77.4	0.563
	DNN^②^	2	16	32	78.6	79.1	79.1	0.196
	SVM				67.0	77.6	77.4	0.149
Mg^2+^	DNN^①^	3	32	32	80.9	82.8	81.9	0.658
	DNN^②^	3	32	32	71.7	85.1	85	0.163
	SVM				77.2	78.9	78.8	0.141

DNN^①^ represents the results of the training dataset, DNN^②^ represents the results of independent testing dataset

It can be seen from the results of the fivefold cross-validation in Table 5 that the undersampling method effectively reduces false positives brought by imbalance between positive and negative sets. The Sn values of two ion ligands reach more than 80.1%, and their prediction performance is more balanced. In the results of the independent testing, Sn value of DNN algorithm reaches 71.7%, Sp and Acc value reached 79.1%, MCC value reached 0.163. In order to compare the prediction performance of DNN algorithm in the undersampling method, we compared the results with the results of SVM algorithm using the undersampling method [13], and the prediction results of independent test of SVM algorithm were also listed in Table 5.

## Discussion

Comparison in Table 3 shows that the evaluation index of DNN algorithm and Ionseq method had the same characteristics, that is, the Sn value was smaller and the SP value was larger, which was related to the fact that the number of negative sets in the data set was much larger than the number of positive sets. However, Sn value and MCC value of DNN algorithm were better, and Sn value of Mg^2+^ was 27.2% higher than Ionseq method. The Sp and Acc values of DNN algorithm were slightly lower than those of Ionseq method.

By comparison, it was found in Table 5 that DNN algorithm was better than SVM algorithm except that the Sn value of Mg^2+^ ligand was slightly lower, and the Sn value of Ca^2+^ ligand was 11.6% higher than that of SVM algorithm. This may be due to the fact that the number of positive sets of Ca^2+^ ligands is more than that of Mg^2+^ ligands, while the DNN algorithm is suitable for big data learning and the SVM algorithm for small sample learning. Therefore, the DNN algorithm has better performance for Ca^2+^ ligands, and the Sn value of the prediction for Mg^2+^ ligand was slightly lower. Therefore, based on undersampling method, we think that the prediction performance of DNN algorithm is better than that of SVM algorithm.

## Conclusion

In this paper, based on protein sequence information, six characteristic parameters were selected and DNN algorithm was used to predict Ca^2+^ and Mg^2+^ ligand binding residues. In order to improve the prediction performance of DNN algorithm, we optimized the number of hidden layers, the number of hidden layer nodes and the batch size of DNN algorithm. With the optimized parameters, the results of fivefold cross-validation were better than those of Ionseq method. At the same time, we also adopted the method of undersampling the data set, and used fivefold cross-validation and independent tests. With the optimized parameters, the independent test results of DNN algorithm were better than those of SVM algorithm. The good prediction results based on the DNN algorithm for predicting Ca^2+^ and Mg^2+^ ligand binding residues are due to the large data set of Ca^2+^ and Mg^2+^ ligand binding residues, which is suitable for the prediction by the DNN algorithm, and the optimized hyper-parameters of the model, which improves the performance of the algorithm.

## Data Availability

The datasets used and analyzed during the current study are available from the corresponding author on reasonable request.

## Abbreviations

DNN: Deep neural network
ANN: Artificial neural network
SVM: Support vector machine
RF: Random forest
Sp: Specificity
Acc: Accuracy of prediction
MCC: Matthew’s correlation coefficient
Sn: Sensitivity
